# Tumor type classification and candidate cancer-specific biomarkers discovery via semi-supervised learning

**DOI:** 10.52601/bpr.2023.230005

**Published:** 2023-04-30

**Authors:** Peng Chen, Zhenlei Li, Zhaolin Hong, Haoran Zheng, Rong Zeng

**Affiliations:** 1 School of Computer Science and Technology, University of Science and Technology of China, Hefei 230026, China; 2 Anhui Key Laboratory of Software Engineering in Computing and Communication, University of Science and Technology of China, Hefei 230026, China; 3 Department of Systems Biology, University of Science and Technology of China, Hefei 230026, China; 4 CAS Key Laboratory of Systems Biology, Shanghai Institute of Biochemistry and Cell Biology, Center for Excellence in Molecular Cell Science, Chinese Academy of Sciences, Shanghai 200031, China; 5 School of Life Science and Technology, ShanghaiTech University, Shanghai 201210, China

**Keywords:** Tumor type classification, Cancer-specific biomarkers, MSSL, Deep learning

## Abstract

Identifying cancer-related differentially expressed genes provides significant information for diagnosing tumors, predicting prognoses, and effective treatments. Recently, deep learning methods have been used to perform gene differential expression analysis using microarray-based high-throughput gene profiling and have achieved good results. In this study, we proposed a new robust multiple-datasets-based semi-supervised learning model, MSSL, to perform tumor type classification and candidate cancer-specific biomarkers discovery across multiple tumor types and multiple datasets, which addressed the following long-lasting obstacles: (1) the data volume of the existing single dataset is not enough to fully exert the advantages of deep learning; (2) a large number of datasets from different research institutions cannot be effectively used due to inconsistent internal variances and low quality; (3) relatively uncommon cancers have limited effects on deep learning methods. In our article, we applied MSSL to The Cancer Genome Atlas (TCGA) and the Gene Expression Comprehensive Database (GEO) pan-cancer normalized-level3 RNA-seq data and got 97.6% final classification accuracy, which had a significant performance leap compared with previous approaches. Finally, we got the ranking of the importance of the corresponding genes for each cancer type based on classification results and validated that the top genes selected in this way were biologically meaningful for corresponding tumors and some of them had been used as biomarkers, which showed the efficacy of our method.

## INTRODUCTION

Cancer biomarker research and tumor type classification provide essential information for cancer diagnosis, estimate prognosis, and targeted therapy. Besides, they are fundamental to advancing personalized medicine (Leary *et al*. 2010). Conventionally, cancer biomarkers are identified in laboratories, where researchers use various methods to measure the level or presence (or absence) of the tumor markers on samples of tumor tissue or bodily fluid. However, experimental identification of cancer biomarkers requires *in vivo* detection and is notoriously costly, time-consuming, and labor-intensive (Novaković 2004). Driven by high-throughput genomic technologies, a large volume of gene expression data has been accumulated, which boosts many gene differential expression analysis technologies in recent years. However, different datasets face the challenge of inconsistent internal variances and label differences. Therefore, exploiting cancer-specific biomarkers and tumor type classification across multiple tumor types and multiple datasets by computational methods is highly needed.

For the discovery of candidate biomarkers on high-dimensional gene expression data, existing published methods can be summarized into these categories: (1) Statistical analysis technologies. Generally, these methods filter differentially expressed genes on pairs of tumor and adjacent normal tissue using parametric test or nonparametric test (Baldi and Long 2001; Jafari and Azuaje 2006), which mainly rely on the statistical characteristics of gene expression data without any learning algorithm. Therefore, these methods are efficient but have poor performance on large-scale data. (2) Machine learning methods. Although the number of measured genes in gene expression data from DNA microarrays is large, only a few underlying gene components account for tumor type classification and these genes are selected as candidate biomarkers through machine learning methods (Díaz-Uriarte and de Andres 2006; Liu *et al*. 2005). However, these methods are unable to detect cancer-specific differentially expressed genes and have limits on multiple datasets. (3) Deep learning methods. Way *et al*. (Way and Greene 2018) proposed a variational autoencoder framework and conducted cancer stratification, and specific activated expression patterns by training it on The Cancer Genome Atlas (TCGA) (The Cancer Genome Atlas Research Network *et al*. 2013) pan-cancer RNA-seq data. Similarly, Dandee *et al*. (Danaee *et al*. 2017) used a stacked denoising autoencoder model to extract deep features of high-dimensional gene expression profiles and performed classification on them. Referencing visualization methods, Lyu *et al*. (Lyu and Haque 2018) embedded gene expression data into 2-D images and made classification based on a deep convolutional neural network. Khoshghalbvash *et al*. (Khoshghalbvash and Gao 2019) constructed an integrative deep neural network to perform classification and feature selection on multi-source genomic data. These deep learning methods show better performance than previous approaches. However, gene expression data is usually high-dimensional and the number of samples for different cancer types is unbalanced. Deep learning framework could gain a good performance on tumor types with a large sample size, as to small samples, the generalization ability becomes weaker. The data volume of the existing single high-quality dataset (such as TCGA) is not enough to fully exert the advantages of deep learning, especially since the sample size of some relatively uncommon tumor types is extremely small. There exist a large number of datasets from different research institutions on the Gene Expression Comprehensive Database (GEO), which are of relatively low quality, with inconsistent internal variances, lack of labels, and other challenges (Barrett *et al*. 2007). Exploiting the use of these datasets to improve the performance of deep learning in gene expression analysis is attractive.

Semi-supervised learning (Chapelle *et al*. 2009) has achieved great success by training a large number of unlabeled samples and a small number of labeled samples together, which avoids wasting data and resources and solves the problem of complex model generalization in supervised learning. Collecting a large-scale dataset with clean labels is still expensive and time-consuming, especially in areas that require expertise. Although high-throughput sequencing technology is developing rapidly, it is not easy to get enough samples for each cancer type in one database and gene expression data from different databases often has batch effects, which does not meet the assumption of homogeneity of variance. Currently, there is no unified mathematical model to integrate gene expression data from different datasets and balance the number of relatively uncommon tumor samples.

To address these limitations, we propose MSSL, a new robust multiple-datasets-based semi-supervised learning model that mixes multiple datasets and applies state-of-the-art data augmentation methods (Xie *et al*. 2019) in supervised learning to the unlabeled samples, the overall process of our method is demonstrated in Scheme 1. Our method was applied to two high-dimensional microarray cancer datasets to identify significant biomarkers, part of TCGA pan-cancer normalized-level3 RNA-seq data as high-quality labeled data and the remaining combined with GEO normalized-level3 RNA-seq data as low-quality unlabeled data. We only used mixed samples and unlabeled samples to train our model to avoid overfitting. To train on mixed samples, we predicted the labels of unlabeled samples through an exponential moving average (EMA) model and progressively increase the batch size of unlabeled samples when mixing samples in the mini-batch, which avoided introducing excessive misinformation, especially at the beginning of training. We applied different augmentation strategies for unlabeled data and calculated consistency loss. Next, with the trained neural network, we followed the idea of Permutation Importance (PI), which was used to measure feature importance in data mining, to explore top genes that contribute most to classification. MSSL outperformed all existing machine learning and deep learning methods on TCGA RNA-seq data and got 97.6% final accuracy. Finally, we validated that the top genes selected in this way were biologically meaningful for corresponding tumors and some of them have been used as biomarkers, which showed the efficacy of our method.

**Figure 1:**
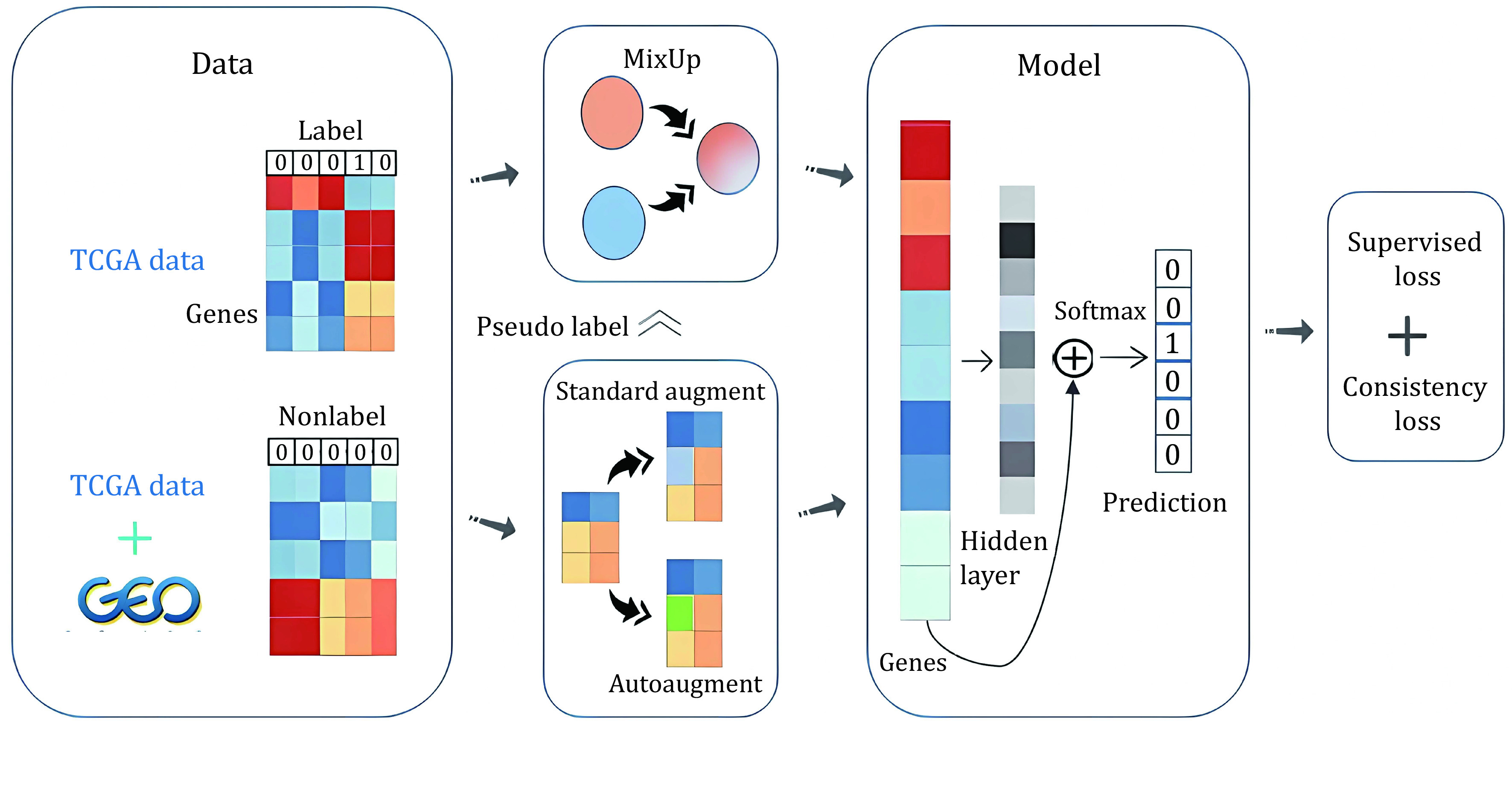
The training process for MSSL. We do MSSL as described in the Section of Materials and Methods. Given our data TCGA and GEO, we do MixUp and Augment, and we introduced the process of guessing labels. We compute supervised loss on mixed samples and consistency loss on augmenting data. Finally, we update the parameters of the training model by minimizing the total loss

The contributions of this study are summarized as follows: (1) We propose a new robust multiple-datasets-based semi-supervised learning model, MSSL, which gets a significant performance leap when applied to TCGA and GEO RNA-seq data compared with previous approaches. (2) We propose an interpolation technique that allows mixed samples to produce higher-quality labels after interpolation to realize the utilization of a large amount of unlabeled data and balance the number of relatively uncommon cancer samples. (3) We explore top genes that contribute most to classification based on the idea of PI and find their relations to the corresponding tumor types, which proves that they can be used as candidate cancer-specific biomarkers.

## MATERIALS AND METHODS

In this section, we first introduce the datasets that are used for training our model. Next, we introduce the algorithms involved in our model which include consistency regularization, interpolation technique, and label guessing. We finally introduce PI, which is used to measure feature importance in data mining. It can relate genes that contribute most to classification to candidate cancer-specific gene biomarkers.

### TCGA dataset

The datasets we applied to MSSL include TCGA and GEO normalized-level3 RNA-Seq gene expression profiles. TCGA (The Cancer Genome Atlas Research Network *et al*.^ 2013^) RNA-Seq gene expression datasets were downloaded from UCSC Xena. Hub (Goldman *et al*. 2018). The data contained 10439 tumor samples concerning 20531 genes for 33 tumor types. The log2(*x* + 1) transformed RNA-Seq by expectation maximization (RSEM) normalized count was used to show the gene-level transcription estimates. To better express the differences between samples, each gene was subtracted from its mean. For better cross-database integration, we downloaded an annotation file of gene and chromosome positions from the National Center for Biotechnology Information (NCBI). According to the positional relationship of genes marked in the table on chromosomes, we rearranged the sequence of the genes and removed around 1000 genes that did not appear in the annotation file. Besides, to balance the classification accuracy and the integrity of the genes, we filtered out the genes whose variance is less than 1.0.

### GEO dataset

GEO which contains about 33,000 human cancer-related gene expression datasets, totaling about 830,000 samples collects experimental data submitted by different research institutions (Barrett *et al*. 2007). Different experiments have different gene chip formats and batch effects between experiments, which results in inconsistent data formats, and the downloaded samples lack label information and cannot be directly used for pan-cancer analysis. To this end, we combined existing toolkits and automated scripting tools (Gautier *et al*. 2004; Carvalho and Irizarry 2010) to design a system for downloading and data preprocessing, and downloaded cancer-related gene expression data in batches from the GEO website. The overall process of our system is demonstrated in Scheme 2. Finally, we got 9979 cancer-related gene expression data samples and processed these samples in the same way as TCGA described above.

**Figure 2:**
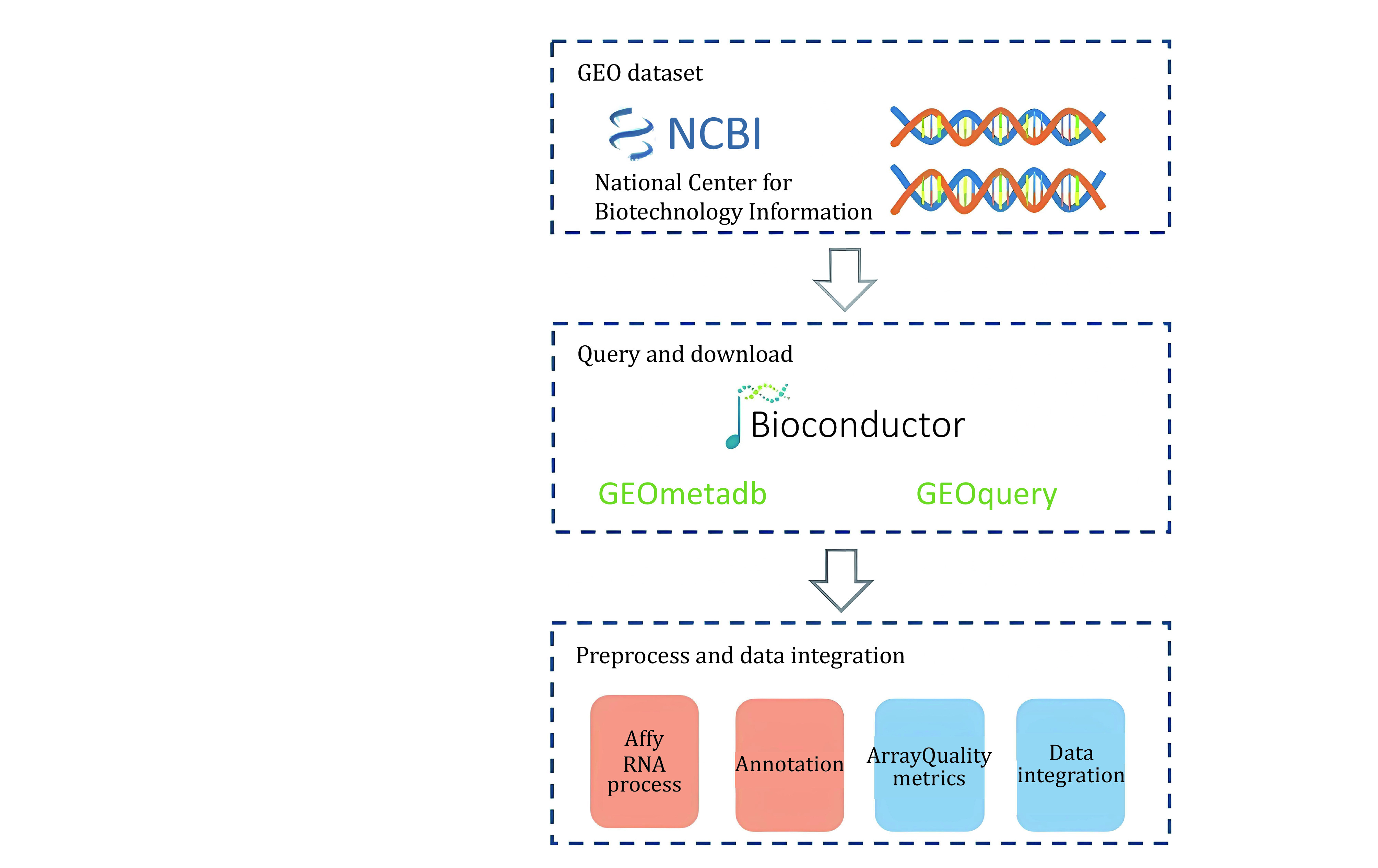
Overview of GEO download

### MSSL model

To learn among multiple cancer datasets, we introduce supervised loss *L*_S_ for labeled samples and consistency loss *L*_C_ between labeled and unlabeled samples and present a multiple-datasets-based semi-supervised learning model MSSL. We utilized a weighting factor λ to balance the supervised loss and the consistency loss and the objective function of MSSL is defined as follows:

1 $ L={L}_{\mathrm{S}} + \lambda {L}_{\mathrm{C}}, $

where *L*_S_ and *L*_C_ are supervised and consistency loss respectively that will be introduced in detail in the next. λ is a weighting factor.

We define the notations for our study. We use $ L=({x}_{b},{y}_{b}^*):b\in (1,\dots ,\mathrm{B}) $ and $ U=({u}_{b,}\;{y}_{b}):b\in (1,\dots ,\mathrm{B}) $ to denote the sets of labeled and unlabeled cancer samples, y^*^ to denote its truth cancer type and *y* to denote its predicted cancer type. We are interested in learning a model *p*_*θ*_(*y*|*x*) to predict *y* based on the inputs *x*, where *θ* denotes the model parameters. Meanwhile, we keep an EMA model $ {p}_{{\theta }'}(y|x) $, where $ {\theta }' $ denotes the EMA model parameters.

### Consistency regularization

Consistency regularization is already one of the most commonly used regularization methods in semi-supervised learning. The main idea of consistency regularization is as follows: for input, even if it is slightly disturbed, the model should output similar predictions. MSSL leverages two kinds of augmentations: standard data augmentation and AutoAugment (Cubuk *et al*. 2018). We applied standard data augmentation once to labeled cancer samples and twice to unlabeled cancer samples, with different strategies. For an unlabeled cancer sample, apply standard augmentation *aug*1 and AutoAugment *aug*2 to generate *aug*1(*u*_*b*_) and *aug*2(*u*_*b*_) and compute the output distribution *p*_*θ*_(*y*|*aug*1(*u*_*b*_)) and *p*_*θ*_(*y*|*aug*2(*u*_*b*_)). Minimize the divergence metric between the two predicted distributions. In our work, we used Kullback-Leibler divergence to calculate consistency loss.

2 $ {D}_{kl}\left(p,q\right)={\sum }_{x}p\left(x\right)\mathrm{log}\left[\frac{p\left(x\right)}{q\left(x\right)}\right], $

where *p* and *q* are the output prediction of the model. Zhang *et al*. (Zhang *et al*. 2017) determined that better data augmentation can outperform consistency regularization as a power component to significantly improve semi-supervised learning.

3 $ {L}_{C}=\frac{1}{\left|B\right|}{\sum }_{b\,=\,1}^{B}{D}_{kl}\left\{{p}_{\theta }\left[y\mid \mathrm{a}\mathrm{u}\mathrm{g}1\left({u}_{b}\right)\right],{p}_{\theta }\left[y\mid \mathrm{a}\mathrm{u}\mathrm{g}2\left({u}_{b}\right)\right]\right\}, $

where the *D*_*kl*_ has been shown in Eq. 2. When using the gradient descent method to update the parameters, the gradient of *p*_*θ*_[*y*|*aug*1(*u*_*b*_)] needs to be stopped.

### Interpolation technique

Normal data augmentation assumes that novel and realistic-looking training data is created by applying a transformation to a single sample without changing its label. Interpolation-based regularization aims to use multiple samples to generate new samples. The main idea is to create new samples by interpolating multiple cancer samples. The representative methods include SMOTE, Sample Pairing and MixUp (Zhang *et al*. 2017), among others. MixUp builds virtual mixed samples by linearly interpolating samples, as shown in Eq. 4.

4 $ \left\{\begin{aligned} &
\lambda \sim \mathrm{Beta}\left(\alpha ,\alpha \right)\\& \tilde {x}=\lambda {x}_{i} + \left(1-\lambda \right){x}_{j}\\&
\tilde {y}=\lambda {y}_{i} + \left(1-\lambda \right){y}_{j}
\end{aligned}\right. ,$

where $ {x}_{i} $ and $ {x}_{j} $ are raw input vectors randomly selected from the cancer samples, $ {y}_{i} $ and $ {y}_{j} $ are one-hot label encodings of cancer types, and λ is a random variable from the $ \mathrm{Beta}\left(\alpha ,\alpha \right) $ distribution. We could obtain an infinite number of mixed cancer samples through simple linear interpolation between samples and then used those mixed samples to train and update the model. MixUp can be seen as a method for data augmentation and causes the model to exhibit linear behavior in the area between different samples. When predicting data outside of training samples, this linear modeling can reduce incompatibility

After data augmentation, given our processed batches *aug*1(*x*_*b*_) and *aug*2(*u*_*b*_). As Eq. 4 shows, the labels of samples are necessary for the MixUp. To generate sample labels, we must predict the pseudo-label for unlabeled samples. We used the outputs of the EMA model as the ‘pseudo label’. Figure 1 shows that EMA model has better performance than the training model after several iterations, so the output of EMA is more accurate than the model. Then we show the detailed process of guessing labels. The parameter of the EMA model $ {\theta }' $ is a weighted average of *θ*, and we define $ {\theta }_{t}' $ as the EMA parameters at training step *t*:

5 $ {\theta }_{t}'=\alpha {\theta }_{t-1}' + \left(1-\alpha \right){\theta }_{t} $

where *α* is a smoothing coefficient hyperparameter. We update the parameter *θ* of the model through backward propagation and update EMA parameter $ {\theta }' $ as Eq. 5.

**Figure 1:**
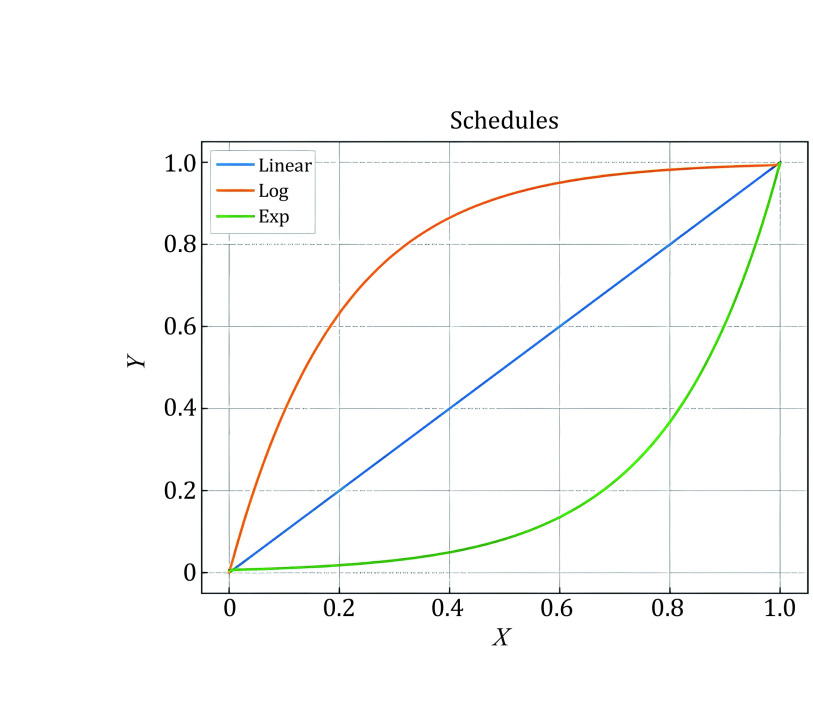
Three schedules of MSSL

Figure 2 shows that the accuracy of pseudo-labels is not high in the early stage of training; if MixUp labeled samples with all batches of unlabeled samples, the label of the mixed sample is not credible, and it will cause too much noise to the training. We simply assume that the quality of the mixed data and the average quality of all samples in mixing are positively correlated. We denote the accuracy of unlabeled samples as *p*(*x*), which grows from 0 to 1 with training in an ideal situation, and the accuracy of labeled samples is 1. Since the number of unlabeled samples is much larger than the number of labeled samples, we set the number of labeled samples to B and the number of unlabeled samples to KB (*k* > 1). Therefore, the average quality of all samples is simply expressed as [1 + *Kp*(*x*)]/(1 + *K*), equivalent to *p*(*x*) + [1 − *p*(*x*)]/(1 + *K*). This means that the smaller the *K* is, the higher the quality of the mixed sample. To prevent overfitting and guarantee the quality of mixed samples, we gradually ramp up the batch size of unlabeled samples from 0 to *K*. The model can smoothly propagate labeled information to unlabeled samples and alleviate the problem of overfitting, which is illustrated in Fig. 3. We set *T* as the total steps of training and *t* as the current step, and we consider three ramp-up schedules to augment the batch size of unlabeled samples. (1) Linear-schedule. $ {\eta }_{t} $ is increased linearly as the training process: $ {\eta }_{t}=\dfrac{t}{T} $ . (2) Log-schedule. $ {\eta }_{t} $ is increased most rapidly at the beginning of the training: $ {\eta }_{t}= 1 -exp\left(-\dfrac{t}{T}*5\right) $. (3) Exp-schedule. $ {\eta }_{t} $ is increased most slowly at the beginning of the training: $ {\eta }_{t}=exp\left(\dfrac{t}{T}  - 1\right) * 5$.

**Figure 2:**
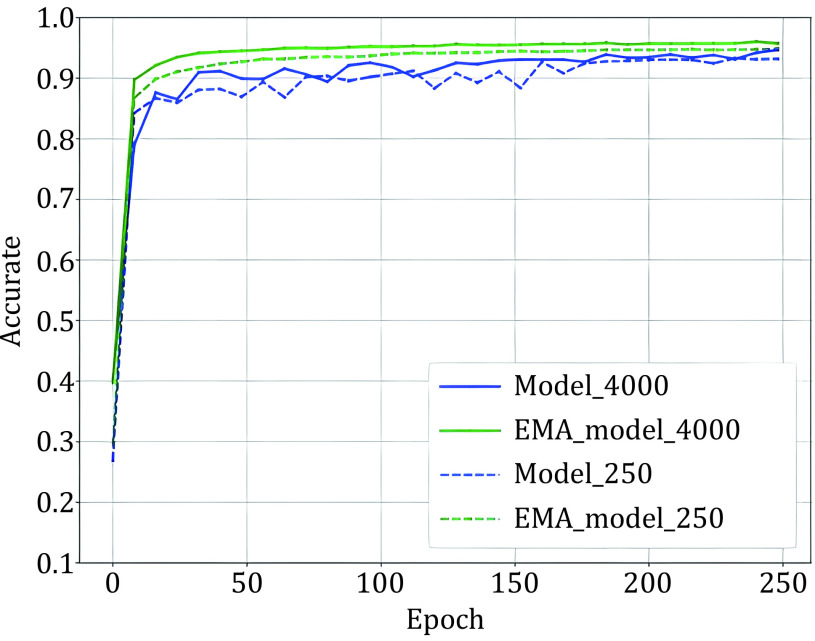
Evaluate the classiﬁcation accuracy of EMA and training model on the test set of TCGA, model-4000 training with 4000 labels samples and model-250 training with 250 labels samples. It shows that the EMA model has better performance after several rounds of epochs until the end of the training. In addition, the performance of the EMA model is more stable

As shown in Fig. 1, the log-schedule is the most suitable schedule when there are only a few labeled samples, and it can alleviate overﬁtting labeled samples by most rapidly increasing the batch size of unlabeled samples. If there are abundant labeled samples, choosing a slower growth strategy (such as exp-schedule or linear-schedule) can help ensure the quality of the mixed label. Finally, we can obtain mixed samples $ {m}_{b} $ and labels $ {y}_{b}^{m} $ by mixup $ aug1\left({x}_{b}\right) $ and $ K{\eta }_{t} $ batches of unlabeled samples $ aug1\left({u}_{b}\right) $ and compute the supervised loss on $ {m}_{b}. $

6 $ H\left(p,q\right)=-{\sum }_{x} p\left(x\right)\mathrm{log}\left[q\left(x\right)\right], $

7 $ {L}_{S}=\frac{1}{\left|B\right|}{\sum }_{b\,=\,1}^{B} H\left[\widetilde {{y}_{b}^{m}},{p}_{\theta }\left(y\mid {m}_{b}\right)\right], $

where *H*(*p,q*) is the cross-entropy between distributions *p* and *q,* $ {y}_{b}^{m} $ is the label of mixed samples, and $ {p}_{\theta }\left(y\right|x) $ is the output of the model.

### Label guessing

As Fig. 2 shows, the EMA model has better and more stable performance. For batches of unlabeled samples $ {u}_{b} $, we need to guess a ‘pseudo label’ using the EMA model’s prediction. Usually, pseudo-labels have two forms: soft labels and hard labels. Hard labels apply Eq. 8 to produce a one-hot probability distribution as pseudo-labels. Sharpen the output distribution to get soft labels by using a low softmax temperature *t* computed as Eq. 9.

8 $ \;{y}^{b}=\mathrm{argmax}\left[{p}_{{\theta }'}\left(y\mid u_b\right)\right], $

9 $ \mathrm{S}\mathrm{h}\mathrm{a}\mathrm{r}\mathrm{p}\mathrm{e}\mathrm{n}(p,t{)}_{i}=\dfrac{\mathrm{exp}\left(\dfrac{{p}_{i}}{t}\right)}{{\displaystyle\sum} _{j=1}^{L} \mathrm{exp}\left(\dfrac{{p}_{i}}{t}\right)}\;. $

Since the mixed label itself has the function of label smoothing, we no longer used confidence masking. To speed up the training of the model, a strategy of asynchronously predicting pseudo-labels can be used. In detail, after every repeating *N* step, we input all unlabeled samples without any data augmentation to the EMA model to predict pseudo labels, then stored the obtained pseudo labels in a dictionary. However, when time permits, it is better to obtain pseudo-labels synchronously.

**Figure 3:**
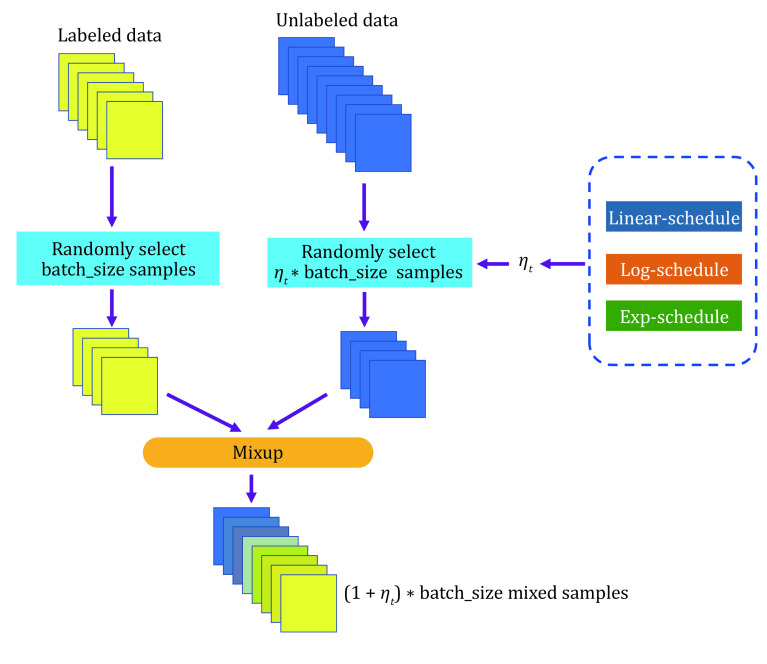
MSSL MixUp. The blue and yellow respectively represent unlabeled data and labeled data

### Model performance evaluation

MSSL model was evaluated by tenfold cross-validation. We calculated the following performance metrics:

10 $ Accuracy=\frac{TP + TN}{TP + TN + FP + FN}, $

11 $ Precision=\frac{TP}{TP + FP}, $

12 $ Recall=\frac{TP}{TP + FN}, $

13 $ F1{\text{-}}score=\frac{2\mathrm{*}Precision\mathrm{*}Recall}{\text{}\text{Precision}\text{ + }\text{Recall}\text{}}. $

### Prediction of candidate cancer-specific biomarkers

To identify candidate genes, we ﬁrst applied TCGA gene expression data to the trained model and obtained the prediction scores of each cancer type. PI is a way to measure feature importance, searching for the features that have the biggest impact on predictions. PI applies to the model that has been ﬁtted, so we only needed to perform ranking importance once in the inference stage. As to each gene feature of each cancer type, we set the single column value to zero and made predictions using the resulting dataset to get the corresponding softmax value of each cancer type. Finally, we calculated the prediction score for each cancer and the absolute value of the softmax value was obtained after resetting a single gene, and arranged the value in descending order, so that we got the ranking of the importance of the corresponding gene for each cancer type. The ranking of each cancer-speciﬁc gene represented the importance of that gene to the classiﬁcation of that type of cancer.

## RESULTS AND DISCUSSION

### Classification

The TCGA gene expression data was divided into the training sets and test sets according to tenfold cross-validation. On each dataset, we set up ﬁve sets of experiments, which were supervised learning of 1000 and 2000 labeled samples, supervised learning of full labeled samples, semi-supervised learning of 1000 labeled samples, and semi-supervised learning of 2000 labeled samples. These ﬁve sets of experiments used the same baseline model.

We used WideResNet-28 (Zagoruyko and Komodakis 2016) as the base model and set the smoothing coefﬁcient hyperparameter α to 0.995 to update the EMA model on all experiments. We trained the model using the SGD optimizer (Nesterov momentum is 0.9) (Loshchilov and Hutter 2016) with an initial learning rate of 0.03 and an L2 weight decay of 0.0005. The batch size of labeled samples was set to 64, the batch size of unlabeled samples was set to 128, and the weight of consistency loss λ was set to 1. Meanwhile, we used the learning rate warmup over the warmup steps from 0 to the initial learning rate and decayed the learning rate with cosine annealing. The experiment included a total of 100 epochs and each epoch performed 1024 iterations. When the number of labeled examples was very limited, the exp-schedule was most suitable to increase the batch size of unlabeled samples. To reduce the time wasted by repeatedly loading the data set, the label samples were expanded several times by copying. We computed the mean and variance of accuracy when training on five different folds of labeled samples.

We compared experimental results with some representative work at present. Kuang *et al*. (Kuang *et al*. 2021) used XGEP to achieve 92.9% accuracy, while Guo *et al*. and Dai *et al*. (Guo *et al*. 2017; Dai *et al*. 2020) proposed Pheg and RWNE respectively and achieved 91.2% and 92% accuracy. Lyu and Haque (Lyu and Haque 2018) achieved 95.5% ﬁnal accuracy based on visualization ideas. As shown in Table 1, MSSL outperformed other methods by a signiﬁcant margin in all TCGA experiments. For example, we achieved an error rate of 4.1% with 2000 labels, which nearly matched the performance of the fully supervised method. Meanwhile, in two experiments with the same number of labeled samples, semi-supervised learning achieved better classiﬁcation accuracy than supervised learning. For example, when using 1000 label samples for training, the classiﬁcation accuracy obtained by semi-supervised learning is 1.0% higher than that of supervised learning, which was 1.4% higher when there are 2000 labeled samples, which fully showed that the semi-supervised learning method can effectively improve the performance of the model, and the improvement was more obvious when the number of label samples was less.

**Table 1:** Performance comparison

Method and samples	Accuracy	Recall	Precision	F1-score
MSSL and TCGA samples	0.968	0.966	0.969	0.967
DNN and TCGA samples	0.945	0.945	0.940	0.942
[10]’s CNN	0.955	0.955	0.955	0.954
XGEP and XGB and all samples	0.929	0.765	0.955	0.749
Pheg and all samples	0.912	0.625	0.969	0.691
RWNE and all samples	0.920	0.509	0.971	0.595
MSSL and TCGA + GEO	0.976	0.978	0.978	0.978

To further improve the performance of the model, especially the performance on relatively uncommon cancer, we increased the GEO RNA-seq data for training. The TCGA was divided into ten labeled sample sets and validation sets based on tenfold cross-validation, the ratio is about 9:1, and the GEO was regarded as an unlabeled sample set. The initial learning rate during training was set to 0.03, which was attenuated by cosine annealing. The batch size of labeled samples was set to 64, and the batch size of unlabeled samples was set to 64. The weight of the consistency loss λ was set to 1. The experiment was trained for a total of 100 epochs, each epoch for 1024 iterations.

As shown in Table 1, in the TCGA dataset for supervised learning and TCGA + GEO for semi-supervised learning two sets of experiments, using GEO dataset for semi-supervised learning of unlabeled samples achieved a higher classiﬁcation accuracy of 97.6%, which indicated that more pan-oncogenes were collected and the obtained analysis results were more reliable.

### Identify and validate candidate cancer-speciﬁc biomarkers

The importance of genes for each cancer type was generated based on the idea of PI. In order to explore whether this ranking of gene importance makes sense, we selected four cancer types, which were breast carcinoma (BRCA), lung squamous cell carcinoma (LUCS), pancreatic adenocarcinoma (PAAD), thyroid carcinoma (THCA), analyzed their corresponding top ten genes (Table 2). And we performed a literature review on the four cancers trying to ﬁnd out the relations between these top genes and tumor types. In BRCA, three of the top ten genes were supported by literature to be related to breast cancer. Its top1 gene GRHL2 high expression is highly correlated with survival rates in all four breast cancer subtypes (Mooney *et al*. 2017), and BAMBI can block transforming growth factor-β (TGF-β) signal transduction from the receptor, and its expression is up-regulated in breast cancer (Wang *et al*. 2015). da Silveira *et al*. (da Silveira *et al*. 2017) revealed that TBX5 controls breast cancer stem cells and forms a mesenchymal or cancer stem cell-like (CSC-like) phenotype. As to LUCS, KRT14, TBX5, DSG3, CDH2, CALML3 and WT1 in the top ten genes were reported to play important roles in LUCS (Zhuo *et al*. 2019; Yang *et al*. 2018). Whereas three related genes in PAAD have been proven to be related to this cancer in the literature. NKX6-1 was reported as a novel immunohistochemical marker for pancreatic and duodenal neuroendocrine tumors (Tseng *et al*. 2015; Cheriyath *et al*. 2011) revealed that the function of IFI27 is mainly related to apoptosis and the tumor suppressor gene FHIT changes in RER(+) pancreatic cancer. Among the top ten genes of THCA, Chen *et al*. (Chen *et al*. 2018) revealed that TSHR is of clinical importance in some thyroid conditions, particularly well-differentiated thyroid carcinoma remnants. Zhu *et al*. (Zhu *et al*. 2015) conﬁrmed that TPO gene variants may be related to thyroid cancer and hypoechoic thyroid nodules. These results showed that TBX5 is related to multiple cancers, however, the other selected genes were not quite the same in different tumor types. It was clear that genes identiﬁed by MSSL were functionally effective, which could be candidate cancer-speciﬁc biomarkers.

**Table 2:** The top ten genes for BRCA, LUCS, PAAD and THCA

Rank	BRCA	LUCS	PAAD	THCA
1	GRHL2	KRT14	NKX6-1	TSHR
2	BAMBI	NACAP1	CASR	CDC7
3	NACAP1	TBX5	NACAP1	TPO
4	DHCR7	CTAGE1	INS	GSTM2
5	TBX5	DSG3	C14orf105	NACAP1
6	GTF2IRD1	CDH2	IFI27	S100A5
7	SDC2	TCF21	FHIT	CD101
8	KIF3A	CALML3	LTBR	VAMP5
9	LMX1B	OSBPL1A	LGI2	KCNJ16
10	TSPYL5	WT1	COL13A1	TGFBRAP1

### Discussion

Gene expression analysis can estimate the likelihood of different cancer for the individual by detecting biomarkers since the expression of some genes in cancer cells will be signiﬁcantly different. However, differential expression analysis based on statistical analysis technology tends to detect shared biomarkers rather than tumor-speciﬁc. Differential expression analysis based on machine learning and traditional deep learning can show excellent performance on common cancers with a large number of samples but has a limited effect on uncommon cancers. MSSL introduces additional datasets and uses semi-supervised learning to perform multi-classiﬁcation on gene expression datasets to detect cancer-speciﬁc differentially expressed genes, which suggests that the semi-supervised learning model can better extract the common and speciﬁc deep features of different cancers, and can balance the learning of each cancer type to achieve better classiﬁcation performance.

## CONCLUSION

In this study, we developed a new robust multiple-datasets-based semi-supervised learning model, MSSL, to make classification of the prevalent forms of cancer and select candidate cancer-speciﬁc biomarkers based on the idea of PI, which is used to measure feature importance in data mining. The results showed that our model got a signiﬁcant performance improvement than previous methods. Moreover, MSSL provides a method to utilize additional datasets to get better generalization performance and achieve good results on uncommon cancer types. In the future, when constructing biological networks based on deep learning, especially when the existing high-quality data is insufﬁcient and other similar data exists, semi-supervised learning has a broad development prospect in cancer transcriptome analysis.

## Conflict of interest

Peng Chen, Zhenlei Li, Zhaolin Hong, Haoran Zheng and Rong Zeng declare that they have no conflict of interest.
